# In-depth analysis of the replication cycle of Orpheovirus

**DOI:** 10.1186/s12985-019-1268-8

**Published:** 2019-12-16

**Authors:** Fernanda Souza, Rodrigo Rodrigues, Erik Reis, Maurício Lima, Bernard La Scola, Jônatas Abrahão

**Affiliations:** 1 Laboratório de Vírus, Departamento de Microbiologia, Instituto de Ciências Biológicas, Universidade Federal de Minas Gerais, Belo Horizonte, Minas Gerais 31270-901 Brazil; 2 Microbes, Evolution, Phylogeny and Infection (MEPHI), Aix-Marseille Université UM63, Institut de Recherche pour le Développement IRD 198, Assistance Publique-Hôpitaux de Marseille (AP-HM), Marseille, France; 3 Institut Hospitalo-Universitaire (IHU)-Méditerranée Infection, Marseille, France

**Keywords:** Orpheovirus, Vermamoeba, Giant viruses, Replication cycle

## Abstract

**Background:**

After the isolation of Acanthamoeba polyphaga mimivirus (APMV), the study and search for new giant viruses has been intensified. Most giant viruses are associated with free-living amoebae of the genus *Acanthamoeba*; however other giant viruses have been isolated in *Vermamoeba vermiformis*, such as Faustovirus, Kaumoebavirus and Orpheovirus. These studies have considerably expanded our knowledge about the diversity, structure, genomics, and evolution of giant viruses. Until now, there has been only one Orpheovirus isolate, and many aspects of its life cycle remain to be elucidated.

**Methods:**

In this study, we performed an in-depth characterization of the replication cycle and particles of Orpheovirus by transmission and scanning electron microscopy, optical microscopy and IF assays.

**Results:**

We observed, through optical and IF microscopy, morphological changes in *V. vermiformis* cells during Orpheovirus infection, as well as increased motility at 12 h post infection (h.p.i.). The viral factory formation and viral particle morphogenesis were analysed by transmission electron microscopy, revealing mitochondria and membrane recruitment into and around the electron-lucent viral factories. Membrane traffic inhibitor (Brefeldin A) negatively impacted particle morphogenesis. The first structure observed during particle morphogenesis was crescent-shaped bodies, which extend and are filled by the internal content until the formation of multi-layered mature particles. We also observed the formation of defective particles with different shapes and sizes. Virological assays revealed that viruses are released from the host by exocytosis at 12 h.p.i., which is associated with an increase of particle counts in the supernatant.

**Conclusions:**

The results presented here contribute to a better understanding of the biology, structures and important steps in the replication cycle of Orpheovirus.

## Background

Giant viruses belong to a complex group of viruses commonly referred to as nucleocytoplasmic large DNA viruses (NCLDVs). The group gained notoriety after the isolation of Acanthamoeba polyphaga mimivirus (APMV), which has large particles (~ 750 nm) capable of infecting amoebae of the genus *Acanthamoeba* [1]. The study and search for new giant viruses has been intensified, and these viruses were uncovered in different samples and environments, considerably expanding our knowledge about their diversity and ubiquity [2, 3]. Most giant viruses such as Mimivirus, Marseillevirus, Pandoravirus and Cedratvirus are associated with free-living amoebae of the genus *Acanthamoeba*; however, other giant viruses have been isolated in *Vermamoeba vermiformis*, such as Faustovirus and Kaumoebavirus [4, 5].

In 2018, a new virus, Orpheovirus IHUMI-LCC2 (hence forward called Orpheovirus), was described that is capable of infecting *V. vermiformis*, which was isolated from rat faecal samples collected in France. These viruses have ovoid-shaped particles, as observed for Cedratvirus, Pandoravirus, and Pithovirus, ranging from 900 to 1100 nm in length and approximately 500 nm in diameter. These viruses are also marked by the presence of a circular double-stranded DNA genome of 1,473,573 bp, encoding 1512 predicted genes of which 57.5% are ORFans [6]. Regarding the predicted genes, it was observed that the best hits were with Pithovirus sibericum, Pithovirus massiliensis and Cedratvirus A11. Furthermore, pan-genomic analysis and phylogenetic reconstructions based on different core genes demonstrated a distant relationship between Orpheovirus and pithoviruses and cedratviruses [6].

Due to its large size, it was proposed that the replication cycle of Orpheovirus was initiated by phagocytosis of viral particles by *V. vermiformis* cells. After particle entry, the genome would be released into the cell cytoplasm through an ostiole located at the apex of the virion. An eclipse phase is established, and then viral factories (VFs) are formed, where new viral particles are assembled. In the final steps of the cycle, the cell cytoplasm is completely filled by new synthesized particles, which are released from the host cell by lysis [6]. Despite the information described in the first proposed model, many steps of the replication cycle and particles of this virus still need to be elucidated.

In the present work, we present an in-depth investigation of the steps of the replication cycle of Orpheovirus. Our data revealed that Orpheovirus induces profound changes in the morphology of *V. vermiformis*, including increased cell motility at some time points of infection. We also provide details about virus entry, VFs formation, organelle recruitment and particle morphogenesis. We also observe that viral particles are released from infected cells both by exocytosis and cell lysis. The data presented here reveal several peculiar characteristics of the life cycle, structure and host interaction of this new giant virus.

## Methods

### Cell culture, viral production, purification and titration

*V. vermiformis* (ATCC CDC19) were cultivated in Peptone Yeast Extract Glucose (PYG) medium supplemented with 0.14 mg/mL penicillin (Sigma-Aldrich, USA), 50 mg/mL gentamicin (Thermo Fisher Scientific, USA), and 2.5 mg/mL amphotericin (Bristol-Myers Squibb, New York, USA) at 32 °C. For Orpheovirus production and purification, ten T175 cm^2^ flasks (Thermo Fisher Scientific, USA) containing 20 × 10^6^ cells in PYG medium were infected with Orpheovirus at a multiplicity of infection (M.O.I.) of 0.01 and incubated for 4 days at 32 °C. The lysate was centrifuged at 1200 x *g* to remove cell debris. Then, the supernatant was collected, added over a 40% sucrose (Merck, Germany) cushion and centrifuged at 36,000 x *g* for 1 h. The pellet was re-suspended in PBS and stored at − 20 °C. Three aliquots of the virus stock were titrated to the 50% end-point and calculated by the Reed-Muench method [7, 8].

### Cytopathic effect, one-step growth curve assays and particle counts

To investigate the cytopathic effect (CPE) of Orpheovirus in *V. vermiformis* cells by optical microscopy, 25 cm^2^ cell culture flasks containing 3 × 10^6^ *V. vermiformis* cells were infected with Orpheovirus at an M.O.I. of 10, incubated at 32 °C and observed at different hours post infection (h.p.i) (1, 3, 6, 9, 12 and 24 h.p.i) for 24 h. Uninfected *V. vermiformis* cells (control) were also observed. A one-step growth curve was constructed using 25 cm^2^ flasks in duplicate at an M.O.I of 10. At different time points (1, 3, 6, 9, 12, 24, 48 and 72 h.p.i), the infected *V. vermiformis* cells and supernatants were collected, titred and calculated using the end point method. We also performed a quantitative polymerase chain reaction (qPCR) assay to quantify the viral genome load targeting the DNA polymerase gene, using oligonucleotide primer sequences Forward 5′- ATGGCGAAATATGCGGAAGGG-3′ and Reverse 5′-TCTTGTGCTCCTAACGCACC-3′. The thermal cycling conditions used were: one cycle at 95 °C for 10 min and 40 cycles at 95 °C for 10 s and 60 °C for 40 s; a melting curve analysis at 95 °C for 15 s, 58 °C for 15 s and a final cycle at 95 °C for 15 s was completed.

To investigate if the particles were released from the host cell by exocytosis, 3 × 10^6^ *V. vermiformis* cells were infected with Orpheovirus at an M.O.I. of 5 and analyses were carried out at the infection times of 3, 6, 9, 12 and 24 h.p.i. Thirty minutes after infection, the monolayer of cells was washed once with PBS and the flasks were filled with 4 mL of PYG medium. After each time point, we separated 12 μL of the supernatant to count the number of released Orpheovirus particles during infection. The particles were observed by light microscopy (OlympusBX41, Japan) under 1000x magnification using a cell counting chamber (Kcell Olen Kasvi, Brazil).

### Transmission and scanning electron microscopy

For the transmission electron microscopy (TEM) assays, the cells were infected at an M.O.I. of 0.01, and when the CPE was observed, we centrifuged the flask content for 10 min at 800 x *g*. The pellet was washed twice with 0.1 M sodium phosphate buffer (pH 7.4) and fixed with 2.5% glutaraldehyde in 0.1 M sodium phosphate buffer for at least 1 h at room temperature. The pellet was then washed twice with 0.1 M sodium phosphate buffer and suspended in the same solution. After, the amoebae were embedded in EPON resin using a standard method, as follows: 2 h of fixation in 2% osmium tetroxide, five washes in distilled water, overnight incubation in uranyl acetate 2% at 2–8 °C, two washes in distilled water, 10 min dehydration in increasing ethanol concentrations (35, 50, 70, 85, 95 and 100%), 20 min incubation in acetone and embedding in EPON resin. Ultrathin sections were subsequently analysed under TEM (Spirit Biotwin FEI-120 kV) at the Center of Microscopy of UFMG, Brazil.

For the scanning electron microscopy (SEM) assays, the cells infected at an M.O.I. of 0.01 were added to round glass blades covered by poly-L-lysine and fixed with 2.5% glutaraldehyde in 0.1 M cacodylate buffer for 1 h at room temperature. Samples were then washed three times with 0.1 M cacodylate buffer and post-fixed with 1.0% osmium tetroxide for 1 h at room temperature. After a second fixation, the samples were washed three times with 0.1 M cacodylate buffer and immersed in 0.1% tannic acid for 20 min. Samples were then washed in cacodylate buffer and dehydrated by serial passages in ethanol solutions with concentrations ranging from 35 to 100%. They were dried at the critical CO_2_ point, transferred onto stubs and metalized with a 5 nm gold layer. The analyses were completed with SEM (FEG Quanta 200 FEI) at the Center of Microscopy of UFMG, Brazil.

### Immunofluorescence assays

For immunofluorescence (IF) analysis, 3 × 10^5^ *V. vermiformis* cells were added to round glass blades and after incubation during 24 h at 32 °C, the cells were infected at an M.O.I. of 5. After 1, 3, 6, 8, 12 and 24 h.p.i., we fixed with acetone for 10 min at − 20 °C. After fixation, cells were stained with polyclonal anti-orpheovirus whole particle antibodies produced in mouse (1:400 diluted in PBS) for 1 h at 37 °C, followed by three rinses with PBS. Cells were then incubated with 3% bovine serum albumin (BSA)-PAS for 30 min, followed by three rinses with PBS. After a 1 h of incubation with anti-mouse secondary antibodies (1:400 diluted in PBS), followed by three rinses with PBS, the cells were also incubated with DAPI Sigma (1:1000 diluted in PBS) for 1 h at room temperature. After three rinses with PBS, the cells were incubated with rodamine-phalloidine (Invitrogen) (1:1000 diluted in PBS) for 1 h at room temperature. Uninfected cells (control) were also fixed and prepared as described above. Fluorescently labelled cells were observed using an Axio Imager Z2-Apotome 2 microscope (Zeiss). The Zen Lite software from Zeiss microscopy was used for image processing at the Center of Microscopy of UFMG, Brazil.

### Membrane inhibitor assays

To evaluate the role of cell membranes in the viral replication cycle, 14 × 10^5^ *V. vermiformis* cells were infected with Orpheovirus at an M.O.I. of 5. Thirty minutes post-infection, the amoebae were washed with PAS and then we added 15 mL of PYG medium maintained at 32 °C. After 1 h, Brefeldin A (BFA), an inhibitor of membrane traffic, was added at a final concentration of 10 μM, and at 8 h post-infection, the amoebae were collected for TEM analysis. Samples were prepared for microscopy as previously described.

## Results

### Characterization of cytopathic effect of Orpheovirus evidenced morphological changes and increased motility of *V. vermiformis*

**Fig. 1:**
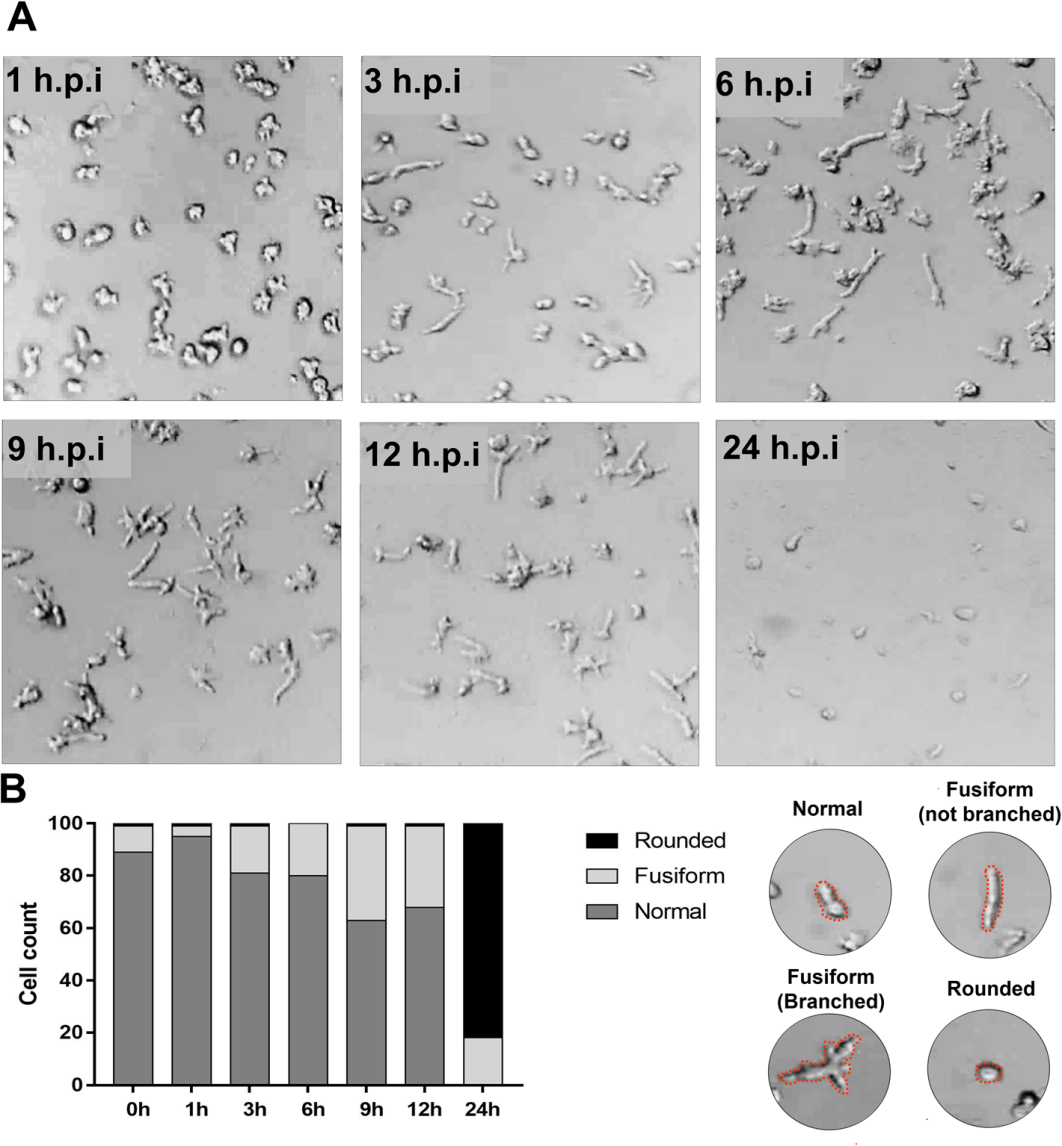
Characterization of cytopathic effect of Orpheovirus. **a** *V. vermiformis* monolayer was infected by Orpheovirus using an M.O.I. of 10 and visualized by light microscopy. Cells became stretched with fusiform shapes at 3 h.p.i., and this effect became more evident at 9 h.p.i. and 12 h.p.i., respectively. At 12 h.p.i., fusiform branched cells were also observed. At 24 h.p.i., most cells observed were rounded; some fusiform cells were also visualized and cell lysis was more evident. **b** Counts of normal, fusiform (branched and not branched) and rounded cells. We observed a decrease of normal cells, while fusiform cells increased at 9 h.p.i., composing ~ 40% of the total infected cells. At 24 h.p.i., most cells were rounded, but some fusiform cells were also noticed (~ 20%)

In order to characterize the CPE of Orpheovirus, *V. vermiformis* cells were infected at M.O.I. of 10 and observed up to 24 h.p.i.. Using optical microscopy, we observed that the cells became stretched into a fusiform shape at 3 h.p.i., and this effect became more evident at 9 h.p.i. and 12 h.p.i., respectively (Fig. 1a). In addition, at 12 h.p.i., some branched fusiform cells were observed (Fig. 1a). At 24 h.p.i., the cells became rounded, cell lysis was more evident and some fusiform cells were visualized (Fig. 1a). We also performed counts of normal (typical morphology of control cells), fusiform (branched and not branched) and rounded cells. We observed a decrease of normal cells, while fusiform cell counts increased at 9 h.p.i., composing ~ 40% of the total infected cells. At 24 h.p.i., most cells were rounded, but some fusiform cells were also observed (~ 20%) (Fig. 1b).

**Fig. 2:**
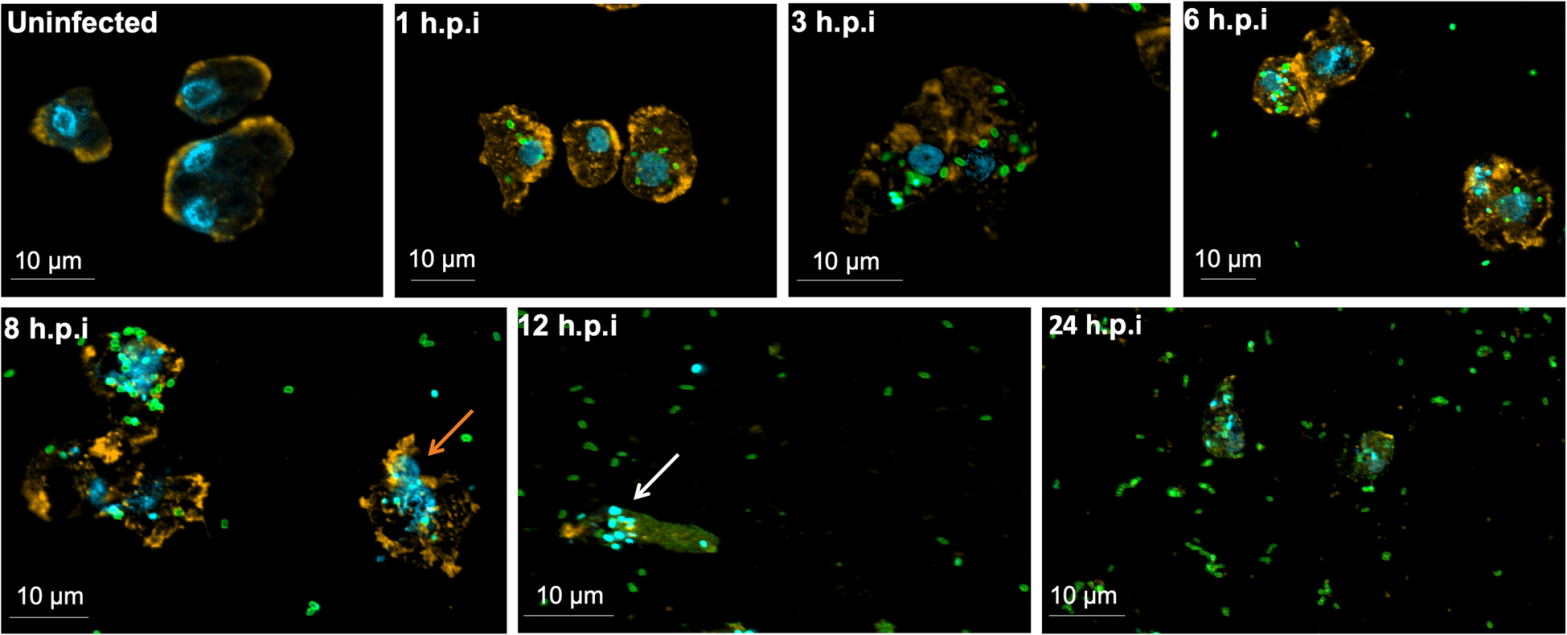
Characterization of the Orpheovirus replication cycle in *V. vermiformis* by IF. *V. vermiformis* monolayer was infected by Orpheovirus at an M.O.I. of 5 and visualized by IF. At early time points, Orpheovirus particles are observed attaching to the surfaces of amoebae. At 3 h.p.i. and 6 h.p.i., respectively, an increase in particle amounts can be visualized within the host cells. At 12 h.p.i., we noticed viral particle polarization at one cell extremity and an increase of viral particles outside the cells. At 24 h.p.i., many cells were rounded, and the large majority of amoebae were already lysed. The viral particles are in green (anti-orpheovirus particle antibody), amoeba cytoskeleton in orange (stained by rodamine-phalloidine) and the nucleus in blue (stained by DAPI). Scale bar, 10 μm


Different time points during infection (M.O.I. of 5) were selected for IF assay. IF assays using anti-orpheovirus primary antibodies at 1 h.p.i. revealed particles being endocytosed by amoebae (Fig. 2). At 3 h.p.i. and 6 h.p.i., respectively, an increase in particle amounts were visualized within the host cells. We also visualized fusiform cells at 12 h.p.i. by IF, and interestingly, we noticed viral particle polarization at one cell extremity and an increasing number of particles outside cells (Fig. 2). At 24 h.p.i., many cells were rounded and the large majority of amoebae were already lysed (Fig. 2). The CPE triggered by Orpheovirus is different from others previously described for Faustovirus, which revealed the formation of plaque forming units, and Tupanvirus, which was characterized by amoebae aggregates called bunches, as well as rounding and lysis in *V. vermiformis* [9, 10]. Furthermore, we observed that Orpheovirus infection induces an increase in the motility of *V. vermiformis* cells, especially those with a fusiform shape. This effect starts at 6 h.p.i. and became more evident at 12 h.p.i. (Additional file 1: Video S1)

**Additional file 1: Video S1** Orpheovirus infection induces an increase in the motility of *V. vermiformis* cells, especially those with a fusiform shape. This effect starts at 6 h.p.i. and became more evident at 12 h.p.i.. This movie is not accelerated.


### Orpheovirus is phagocytized, forms electron-lucent viral factories and induce cytoplasmic changes involving different organelles

**Fig. 3:**
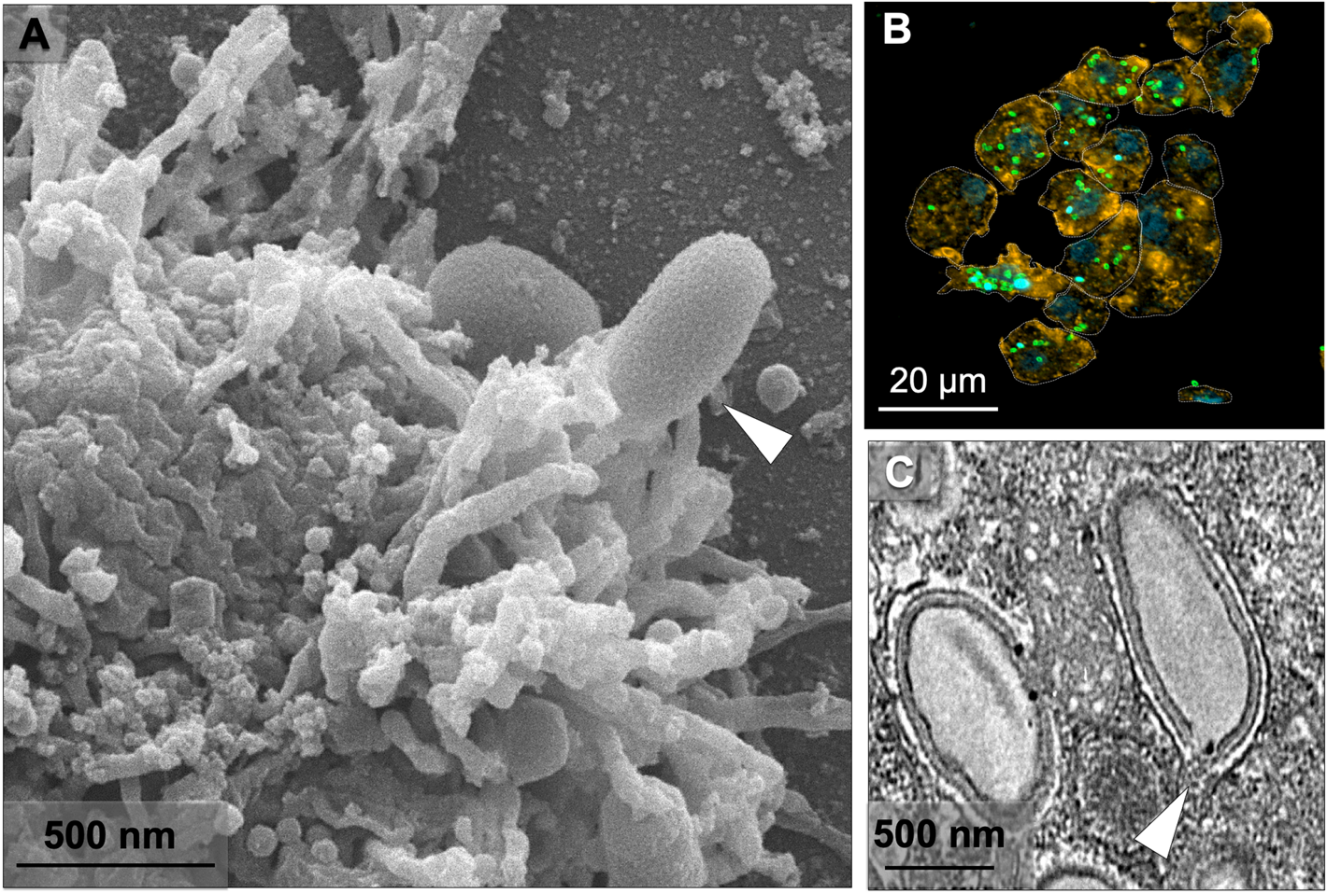
Orpheovirus entry in *V. vermiformis* cells. **a** SEM at 24 h.p.i showing an Orpheovirus particle attached to a *V. vermiformis* cell (white arrow). **b** IF assays at 1 h.p.i. revealed that more than one particle is able to penetrate the host cell. **c** TEM images at 24 h.p.i demonstrate that the internal particle content is released into the cell cytoplasm through an ostiole located at the apex of the viral particle (white arrow)

Due to the large size of Orpheovirus particles (~ 1.1 μm), it was proposed that their entry into *V. vermiformis* cells would occur by phagocytosis, as previously described for other giant viruses, such as Pandoravirus, Mimivirus and Cedratvirus [3, 11, 12]. During early steps of the replication cycle, we visualized, by SEM, the formation of pseudopods in contact with Orpheovirus particles at the cell surface (Fig. 3a). This suggests that phagocytosis is the entry strategy used by Orpheovirus, as previously suggested [6]. IF analyses at 1 h.p.i. demonstrated that more than one particle is able to penetrate the host cell (Fig. 3b). After entry, it was observed, by TEM, that the internal particle content is released into the cell cytoplasm through an ostiole located at the apex of the viral particles (Fig. 3c).

**Fig. 4:**
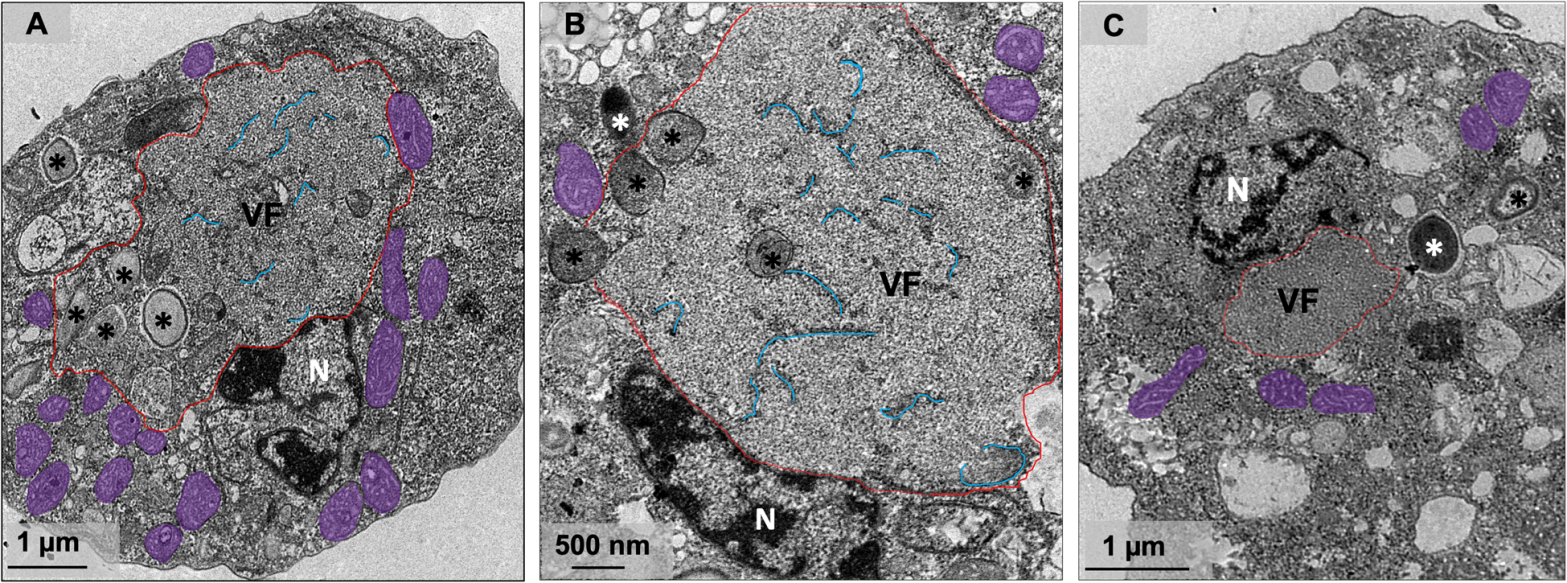
Electron-lucent viral factory and treatment with membrane trafficking inhibitor. **a-b** TEM image at 24 h.p.i shows that Orpheovirus present an electron-lucent VF (contoured in red and in detail), which occupy a large part of the host and is observed in perinuclear regions. We also observed membrane recruitment inside the VF (blue), and the host nucleus remains present during the infection. **c** Treatment with BFA impacts both the formation of VFs and morphogenesis of new particles. VF: viral factory; N: nucleus; Mitochondria are highlighted in purple; *viral particles

**Fig. 5:**
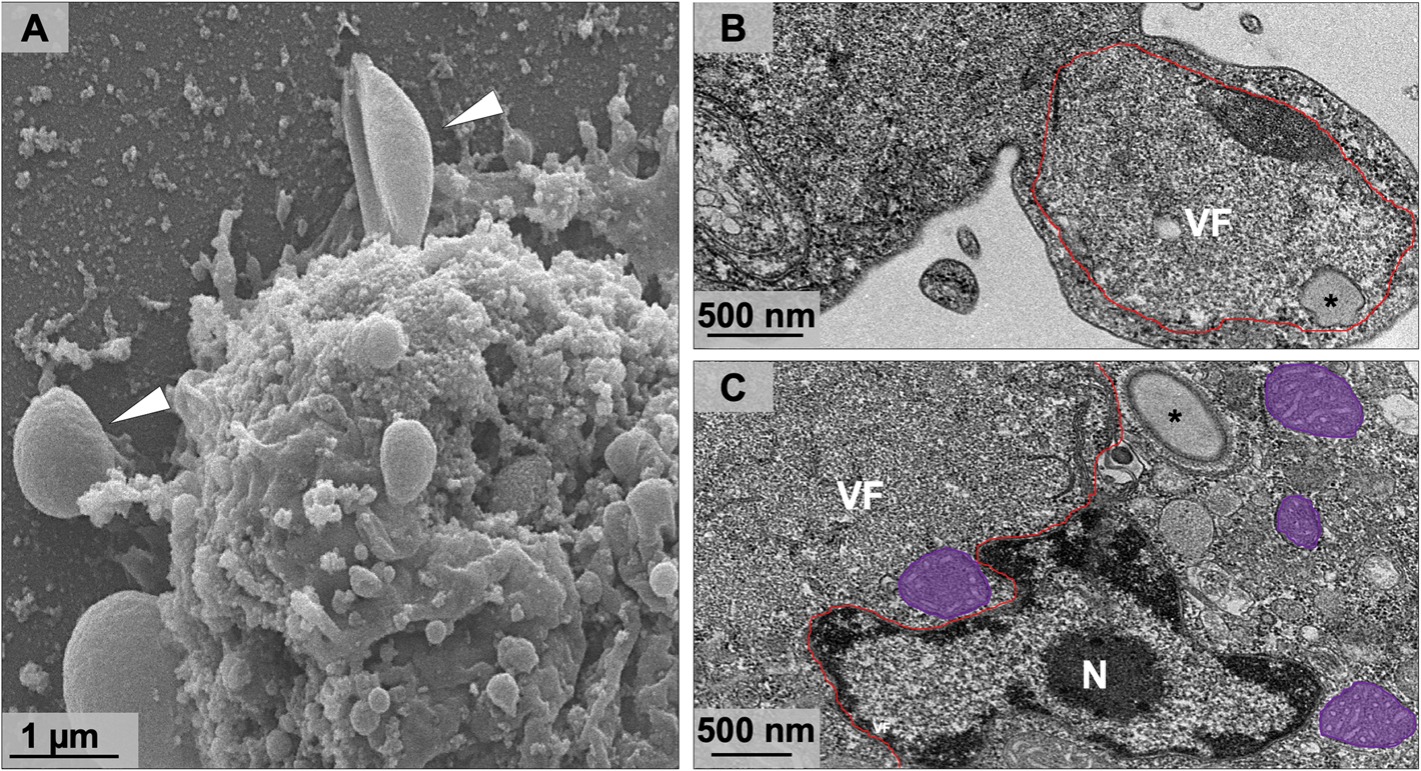
Orpheovirus infection induces bleb formation. **a** SEM at 24 h.p.i showing bleb formation (white arrow) induced by Orpheovirus infection. **b** TEM image at 24 h.p.i revealed the formation of VFs with presence of particles inside blebs in advanced steps of the viral replication cycle. **c** Mitochondrial recruitment was also observed in peripheral regions and inside the VFs. Mitochondria are highlighted in purple. VF: viral factory; N: nucleus; *viral particles

As previously described in other giant viruses, Orpheovirus morphogenesis occurs in subcellular microenvironments called VFs, which are located in the host cell’s cytoplasm. Similar to Cedratvirus and Pandoravirus VFs, Orpheovirus VFs are large electron-lucent areas, which occupy a large part of the host cell (Fig. 4a and b), and do not exhibit well-defined zones as observed for mimiviruses [11]. We also visualized, by TEM, the formation of VFs in perinuclear regions, and unlike those described for pandoraviruses, the host nucleus remains present during the infection (Fig. 4a–c) [3]. Interestingly, the formation of VFs with the presence of particles inside blebs in advanced steps of the viral replication cycle were evidenced by TEM images (Fig. 5a and b). Induction of bleb formation has also been described during Cedratvirus getuliensis infections in *A. castellanii* cells, but this event requires further investigation [12].

Mitochondrial recruitment was also observed in peripheral regions and inside VFs (Fig. 5c), as well as membrane recruitment (Fig. 4a and b). The treatment of infected cells with BFA, a membrane trafficking inhibitor, at 8 h.p.i., affected both the formation of VFs and morphogenesis of new particles (Fig. 4c).

### Morphogenesis dynamics of Orpheovirus particles

**Fig. 6:**
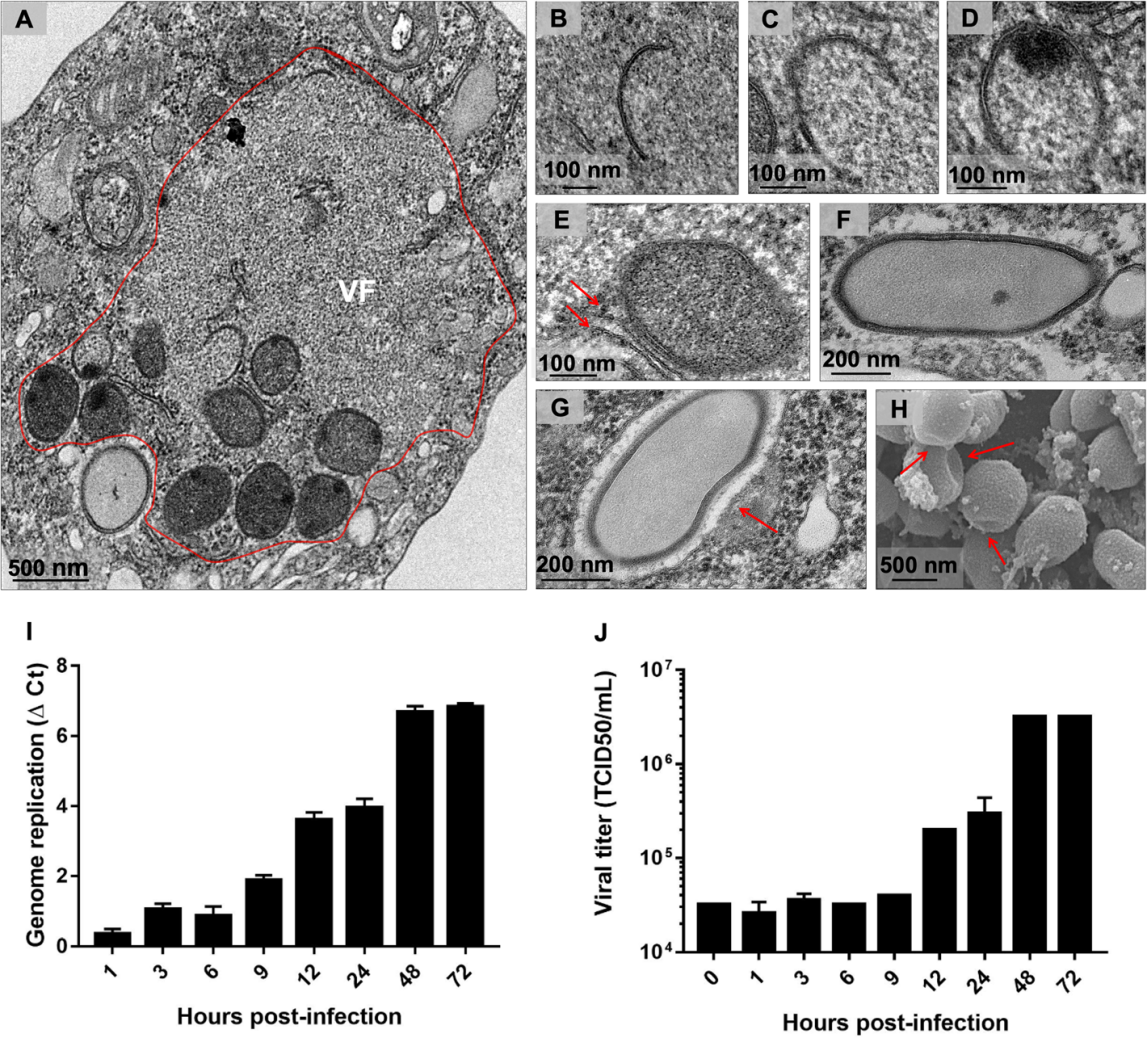
Morphogenesis dynamics of Orpheovirus particles. **a** TEM images at 24 h.p.i of asynchronous cycle of Orpheovirus evidencing the formation of VFs that presented particles in different maturation stages. **b** First discernible viral structures showing a crescent-shaped capsid precursor. **c** and **d** These structures extend and are filled by the internal content. **e** Recruitment of membranes in the periphery of the particles under assembly is also observed (red arrow). **f** Complete closure of the capsids and formation of mature particles. **g** and **h** Both by TEM and SEM images, it was evidenced that the mature particles have a flattening on one side (red arrow). **i** and **j** Orpheovirus one-step growth curve at an M.O.I. of 10

With analysis of TEM images of asynchronous infection of Orpheovirus (M.O.I. of 0.01), the formation of VFs that presented particles in different maturation stages was visualized (Fig. 6a), which obtained more information about the morphogenesis of new particles. The morphogenesis of Orpheovirus starts with the formation of electron-dense semicircular structures named crescents, as observed for other giant viruses, such as Faustovirus and Cedratvirus [9, 12]. These structures extend and are filled by the internal content until the formation of a mature particle (Fig. 6b–f). Recruitment of sheet-like structures in the periphery of the particles under assembly was also observed (Fig. 6e) and may be important for particle formation, which seems to be composed of several layers of proteins and membranes. Both by TEM and SEM images, it was evidenced that the mature particles have a flattening on one side (Fig. 6g and h).

In order to chronologically analyse viral genome replication and infectious particle formation, one-step growth assays were performed. An increase in genome amplification was observed at 9 h.p.i., and infectious particle detection increased at 12 h.p.i. (Fig. 6i–j). At 48 h.p.i., Orpheovirus propagation reached a plateau (Fig. 6i–j). The data corroborate those previously described [6], which suggested that the cycle of Orpheovirus in *V. vermiformis* is slower compared to other giant viruses, during approximately 30 h.p.i.

**Fig. 7:**
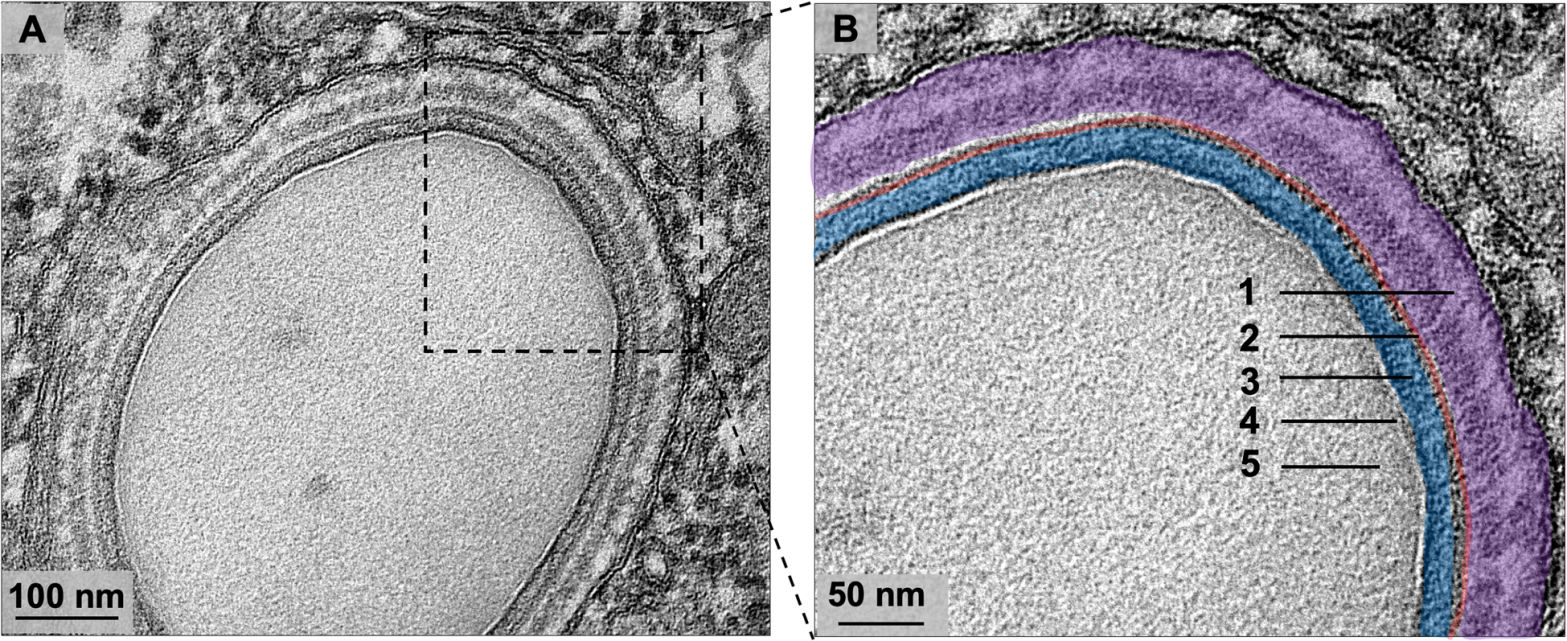
Orpheovirus ultrastructure**. a** and **b** The analyses by TEM revealed that mature particles present a small fibril layer (1, purple), an outer layer (2, red), a capsid layer (3, blue) and an inner membrane (4, white) that involve the core of the particle (5, grey)

**Fig. 8:**
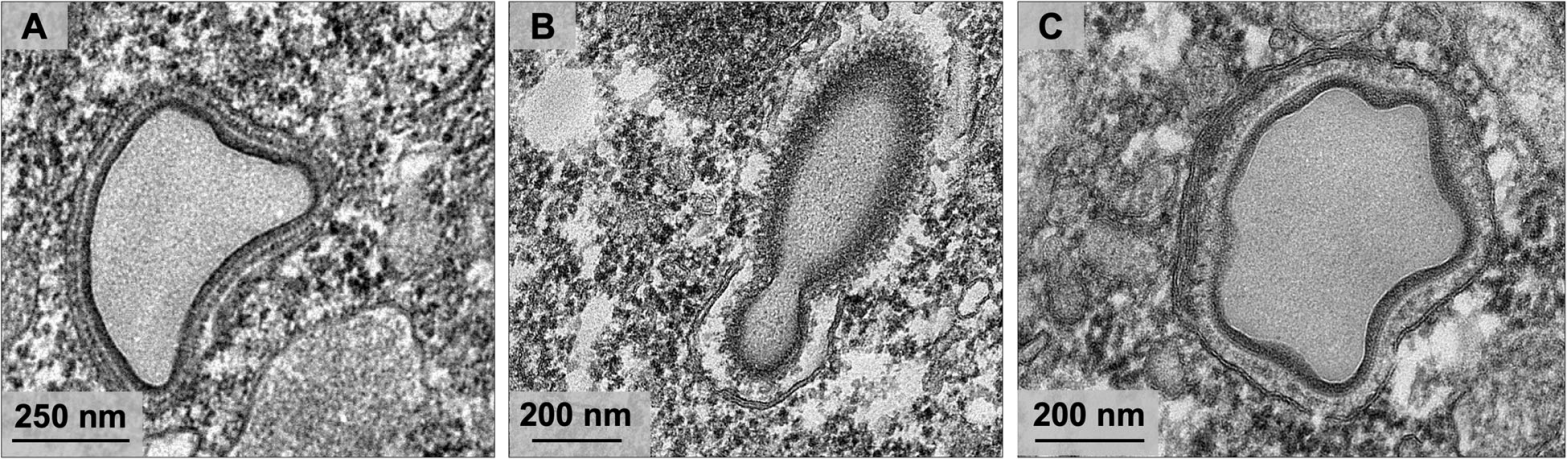
Defective particles observed during Orpheovirus multiplication. **a–c** TEM analyses evidenced the presence of defective particles in different formats, even in infections with low M.O.I. and without the presence of virophage

In addition, it was observed that Orpheovirus particles have a smaller fibril layer compared to those observed in mimiviruses (Fig. 7a and b) [13]. The analyses also revealed that mature particles were present in an outer layer, a capsid layer and an inner membrane that involve the core of the particle (Fig. 7a and b). It is noteworthy that, even in infections with low M.O.I., the presence of defective particles in different formats was evidenced (Fig. 8a-c). This finding suggests that malformed particles occur naturally, which is similar to other giant viruses [6, 10, 11].

### Orpheovirus particles are released from the cell by lysis and exocytosis

**Fig. 9:**
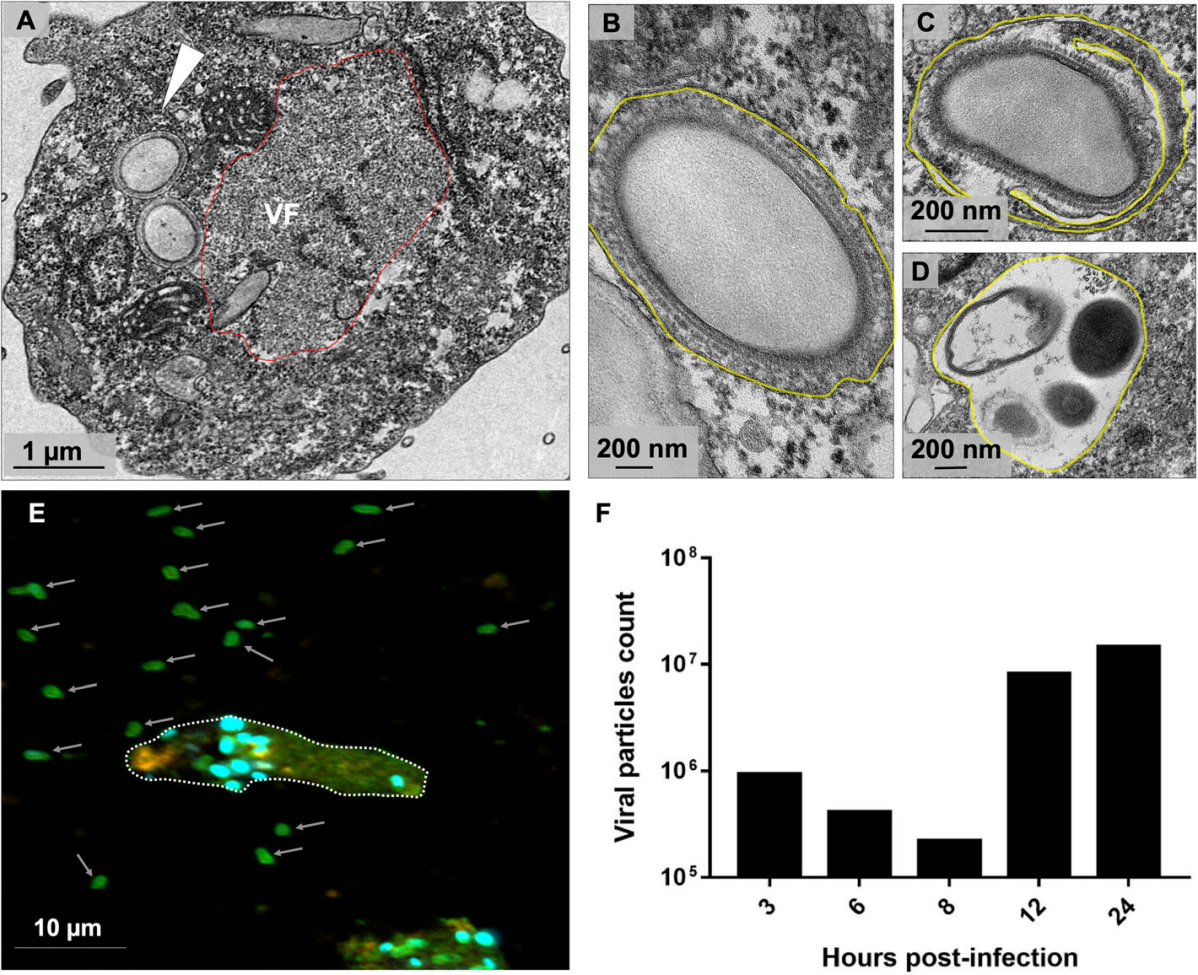
Orpheovirus particles are released from the cell by exocytosis. **a** TEM image at 24 h.p.i evidencing new particles in peripheral regions in the host cell cytoplasm (white arrow). **b** and **c** Some particles were observed involved by one or more membranes (highlighted in yellow). **d** More than one particle in the same vacuole were evidenced. **e** IF assays at 12 h.p.i showing fusiform cells and an increase of particles outside the host cells released by exocytosis (white arrows). **f** Count of Orpheovirus particles in the supernatant over the replication cycle (M.O.I. = 5)

**Fig. 10:**
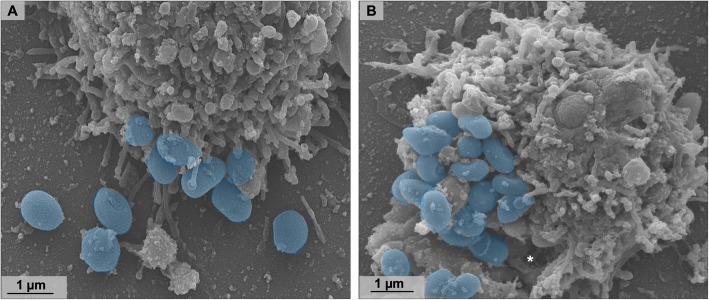
Orpheovirus particles are released from the cell both by exocytosis and lysis. **a** SEM at 24 h.p.i showing particles released from the cell by exocytosis (highlighted in blue). **b** SEM at 24 h.p.i showing particles released from the cell by lysis (highlighted in blue). Asterisk shows damage on cell surface, where particles seem to be released

After VF formation, expansion of the crescents, and complete maturation, new particles were visualized in peripheral regions in the host cell’s cytoplasm (Fig. 9a), and some are involved with membranes. Furthermore, the presence of one or more particles in the same vacuole was observed, which may have more than one membrane (Fig. 9b–d). The presence of viral particles within vacuoles has also been reported for other giant viruses such as Pithovirus and Pandoravirus, suggesting that particles are released from host cells by exocytosis [3, 14, 15]. Biological assays, including cell and particle counts in the supernatant of infected amoebae were performed over the replication cycle (M.O.I. = 5). These analyses revealed that Orpheovirus particles can be detected in the supernatant of infected cells, even at times when no cell lysis is observed (Fig. 9e and f). An increase in particle numbers was noted in the supernatant at 12 h.p.i. In addition, IF analyses performed at different times throughout the cycle also demonstrated an increase in the number of particles outside the cells at 12 h.p.i. and 24 h.p.i., respectively (Fig. 9e and Fig. 2), which reinforces exocytosis as an alternative strategy for host cell particle release (Fig. 10a), although cell lysis was also demonstrated by SEM images (Fig. 10b).

## Discussion

Giant viruses have notable differences in their replication cycles and structures of their virions. After the discovery of this group of viruses, studies have been conducted to increase the knowledge of their biology and host interaction through investigations related to the life cycle of these organisms [11, 16, 17]. This study presents an in-depth description of the stages of the Orpheovirus life cycle, providing information about the entry, morphogenesis, release, structural characteristics and CPE triggered by this virus in its host cells. Other giant viruses are capable of inducing morphological changes in *V. vermiformis*, triggering amoebae aggregates called bunches, as described for Tupanvirus [10], and formation of plaque forming units evidenced for Faustovirus [9], as well as rounding and cell lysis. The morphological changes triggered by the Orpheovirus are different from those previously described, presenting cells with fusiform shapes and some branched cells. It is known that most giant viruses trigger cell rounding, which prevents the formation of pseudopods, and consequently decrease the motility of amoebae. Interestingly, in addition to morphological changes, we observed an increase in *V. vermiformis* cell motility never described before. Although speculative, one hypothesis is that this increased motility would be a strategy that would facilitate the infection of neighbouring cells, leading to an increased spread of viral particles.

The strategy of phagocytosis entry has often been described for other giant viruses, demonstrated through biological assays with pharmacological inhibitors for APMV and Cedratvirus getuliensis, which showed a considerable decrease in viral titres [11, 12]. Considering the fact that Orpheovirus particles are large (900–1100 nm), it was previously proposed that particle penetration would occur by phagocytosis [6]. After observing several SEM images, we noticed the formation of pseudopods in contact with Orpheovirus particles at the surface of host cells. Due to this, we reinforce the hypothesis that phagocytosis would be exploited as an entry strategy, although other entry mechanisms should not be discarded. Once viruses enter cells, the internal particle content is released into the cell cytoplasm through an ostiole located in its apical region. This mechanism is also described for other giant viruses that have particles with similar morphology and are phylogenetically related to Orpheovirus [12, 14].

Unlike VFs described for mimiviruses, which present well-defined zones [11], VFs visualized during Orpheovirus infections are large electron-lucent areas located in perinuclear regions, where the morphogenesis of new particles occurs. Different than that previously observed for pandoraviruses, the nucleus remains during viral infection [3]. In addition, mitochondrial recruitment was observed in peripheral regions of VFs. Membrane recruitment was also observed inside VFs and is suggested to be important for the morphogenesis and vesicle formation phases for further particle exocytosis. Nevertheless, the origin of the recruited membranes needs to be investigated. Marseillevirus vesicles have recently been shown to consist of endoplasmic reticulum membranes, and after treatment with inhibitors, there was a decrease in formation of new particles and vesicles [17]. Also for Orpheovirus, we observe that the treatment with brefeldin impacts VF formation and morphogenesis. The morphogenesis of Orpheovirus particles occurs within the VFs, beginning with the formation of electron-dense semicircular structures called crescents. The formation of these structures is described for other giant amoeba viruses as well as other viruses belonging to the NCLDV group, such as Poxvirus [18]. These crescents become thicker, and throughout the cycle, they are filled with the inner particle content and become more electron-dense until completely closed.

Although Orpheovirus particles present an ovoid-shape, similar to pandoraviruses, cedratviruses, and pithoviruses, they have a distinct structure. They have a fibril layer that is smaller compared to other giant viruses, such as mimiviruses, that have fibrils, and despite scarce information about their constitution, they may be related to host cell adhesion, as demonstrated for mimiviruses [13]. Unlike Mimivirus, no specific area in which fibrils could be acquired was observed in VFs during Orpheovirus infection, and thus we hypothesized that they would be acquired within VFs [11].

Moreover, it was observed that, in addition to fibrils, the particles have an outer layer to the capsid layer and an inner membrane surrounding the core. The presence of one or more particles involved by membranes is described for Cedratvirus getiliensis and Pithovirus sibericum, suggesting that they would be released from the host cell by exocytosis [12, 14]. Although cell lysis has been demonstrated by SEM images and is described as the main strategy for viral progeny release, it is believed that exocytosis is an alternative mechanism used by Orpheovirus. A significant increase in particle counts of the supernatant was noted at 12 h.p.i., with no decrease in cell counts. In addition, by IF microscopy images throughout the cycle, we visualized an increase in the number of particles outside the cells at 12 h.p.i. and 24 h.p.i., respectively.

One-step growth assays showed an increase in viral titres at 12 h.p.i. and increased viral genome amplification by qPCR at 9 h.p.i., reaching a plateau at 48 h.p.i. by both qPCR analysis and titration. These data corroborate those previously proposed [6], which showed a replication cycle duration of approximately 30 h. Other giant viruses, such as Tupanvirus, Kaumoebavirus and Faustovirus, capable of infecting *V. vermiformis* have also been shown to have a longer duration multiplication cycle [4, 5, 10] compared to giant viruses associated with amoebae of the genus *Acanthamoeba*. This suggests that the replication cycle duration profile of these viruses might be related to their host. Altogether, this work brings valuable information concerning the replication cycle, structure and CPE triggered by Orpheovirus, therefore contributing to enhance our understanding about this new giant virus. Future studies applying alternative methods will help to fill some gaps, especially concerning the virus-host interaction.

## Conclusions

In summary, the data presented here reveal several peculiar characteristics of the life cycle, structure, CPE and host interaction contributing to improve our understanding about this new giant virus.

## Data Availability

All relevant information is provided in this current manuscript. If required, the data presented in this work can be shared by e-mail.

## Abbreviations

APMV: Acanthamoeba polyphaga mimivirus
BFA: Brefeldin A
CPE: Cytopathic effect
h.p.i: hours post infection
IF: Immunofluorescence
M.O.I: Multiplicity of infection
qPCR: quantitative polymerase chain reaction
SEN: Scanning electron microscopy
TEM: Transmission electron microscopy
VFs: Viral factories

## References

[1] La Scola B, Audic S, Robert C, Jungang L, De Lamballerie X, Drancourt M, Birtles R, Claverie JM, Raoult D (2003). A giant virus in amoebae. Science.

[2] Pagnier I, Reteno DGI, Saadi H, Boughalmi M, Gaia M, Slimani M, Ngounga T, Bekliz M, Colson P, Raoult D, La Scola B (2013). A decade of improvements in mimiviridae and marseilleviridae isolation from amoeba. Intervirology.

[3] ACDS PA, Victor de Miranda Boratto P, RAL R, Bastos TM, Azevedo BL, Dornas FP, Oliveira DB, Drumond BP, Kroon EG, Abrahão JS (2018). New isolates of Pandoraviruses: contribution to the study of replication cycle steps. J Virol.

[4] Reteno DG, Benamar S, Khalil JB, Andreani J, Armstrong N, Klose T, Rossmann M, Colson P, Raoult D, La Scola B (2015). Faustovirus, an Asfarvirus-related new lineage of Giant viruses infecting amoebae. J Virol.

[5] Bajrai LH, Benamar S, Azhar EI, Robert C, Levasseur A, Raoult D, La Scola B (2016). Kaumoebavirus, a new virus that clusters with Faustoviruses and Asfarviridae. Viruses.

[6] Andreani J, Khalil JYB, Baptiste E, Hasni I, Michelle C, Raoult D, Levasseur A, La Scola B (2018). Orpheovirus IHUMI-LCC2: a new virus among the giant viruses. Front Microbiol.

[7] Reed LJ, Muench H (1938). A simple method of estimating fifty per cent endpoints. Am J Epidemiol.

[8] Abrahão JS, Oliveira GP, Ferreira Da Silva LC, Dos Santos Silva LK, Kroon EG, La Scola B (2016). Mimiviruses: Replication, purification, and quantification. Curr Protoc Microbiol.

[9] Borges I, Rodrigues RAL, Dornas FP, Almeida G, Aquino I, Bonjardim CA, Kroon EG, La Scola B, Abrahão JS (2019). Trapping the Enemy: Vermamoeba vermiformis Circumvents Faustovirus Mariensis Dissemination by Enclosing Viral Progeny inside Cysts. J Virol.

[10] Silva LCF, Rodrigues RAL, Oliveira GP, Dornas FP, La SB, Kroon EG, Abrahão JS (2019). Microscopic analysis of the tupanvirus cycle in vermamoeba vermiformis. Front Microbiol.

[11] ACDSP A, RAL R, Oliveira GP, Andrade KR, Bonjardim CA, La Scola B, Kroon EG, Abrahão JS (2017). Filling knowledge gaps for Mimivirus entry, Uncoating, and morphogenesis. J Virol.

[12] Silva LKDS, Andrade ACDSP, Dornas FP, Rodrigues RAL, Arantes T, Kroon EG, Bonjardim CA, Abrahaõ JS (2018). Cedratvirus getuliensis replication cycle: an in-depth morphological analysis. Sci Rep.

[13] Rodrigues RAL, dos Santos Silva LK, Dornas FP, de Oliveira DB, Magalhães TFF, Santos DA, Costa AO, de Macêdo FL, Magalhães PP, Bonjardim CA, Kroon EG, La Scola B, Cortines JR, Abrahão JS (2015). Mimivirus fibrils are important for viral attachment to the microbial world by a diverse glycoside interaction repertoire. J Virol.

[14] Legendre M, Bartoli J, Shmakova L, Jeudy S, Labadie K, Adrait A, Lescot M, Poirot O, Bertaux L, Bruley C, Couté Y, Rivkina E, Abergel C, Claverie JM (2014). Thirty-thousand-year-old distant relative of giant icosahedral DNA viruses with a pandoravirus morphology. Proc Natl Acad Sci U S A.

[15] Legendre M, Fabre E, Poirot O, Jeudy S, Lartigue A, Alempic JM, Beucher L, Philippe N, Bertaux L, Christo-Foroux E, Labadie K, Couté Y, Abergel C, Claverie JM (2018). Diversity and evolution of the emerging Pandoraviridae family. Nat Commun.

[16] Mutsafi Y, Shimoni E, Shimon A, Minsky A (2013). Membrane assembly during the infection cycle of the Giant Mimivirus. PLoS Pathog.

[17] Arantes TS, Rodrigues RAL, dos Santos Silva LK, Oliveira GP, de Souza HL, Khalil JYB, de Oliveira DB, Torres AA, da Silva LL, Colson P, Kroon EG, da Fonseca FG, Bonjardim CA, La Scola B, Abrahão JS (2016). The large Marseillevirus explores different entry pathways by forming Giant infectious vesicles. J Virol.

[18] Maruri-Avidal L, Domi A, Weisberg AS, Moss B (2011). Participation of Vaccinia virus L2 protein in the formation of crescent membranes and immature Virions. J Virol.

